# The combined effect of green tea and acute interval sprinting exercise on fat oxidation of trained and untrained males

**DOI:** 10.20463/jenb.2016.03.20.1.1

**Published:** 2016-03-31

**Authors:** Daniel E Gahreman, Yati N Boutcher, Sonia Bustamante, Stephen H Boutcher

**Affiliations:** 1 Department of Exercise and Sport Science, Charles Darwin University, Northern TerritoryAustralia; 2 School of Medical Sciences, University of New South Wales, SydneyAustralia; 3 Bioanalytical Mass Spectrometry Facility, University of New South Wales, SydneyAustralia

**Keywords:** Tea Catechins, Interval Sprinting Exercise, Epinephrine, Norepinephrine

## Abstract

**[Purpose]:**

This study investigated the combined effect of green tea and acute interval sprinting exercise on fat oxidation of trained and untrained males.

**[Methods]:**

Fourteen trained and 14 untrained males ingested one capsule containing either green tea or cellulose with breakfast, lunch, and dinner, 24 hours before two exercise sessions. A fourth capsule was consumed 90 minutes before exercise after overnight NPO (nil per os). Participants performed a 20-minute interval sprinting cycling protocol, consisting of repeated bouts of 8-seconds of sprint cycling (at 65% of maximum power output) and 12-seconds of recovery (at 25% of maximum power output), followed by 75 minutes of post-exercise recovery.

**[Results]:**

Fat oxidation was significantly greater in the resting condition after green tea ingestion (p < 0.05) compared with the placebo. Fat oxidation was also significantly increased post-exercise in the green tea, compared with the placebo condition (p < 0.01). During and after exercise the plasma glycerol levels significantly increased in both groups after green tea consumption and were significantly higher in the untrained group compared with the trained group (p < 0.05). Compared with the placebo, the plasma epinephrine levels were significantly higher for both groups in the green tea condition during and after exercise, however, norepinephrine levels were only significantly greater, p < 0.05, during and after exercise in the untrained group.

**[Conclusion]:**

Green tea significantly increased resting and post-exercise fat oxidation and also elevated plasma glycerol and epinephrine levels during and after interval sprinting. Glycerol and norepinephrine levels during interval sprinting were significantly higher in the untrained group compared with the trained group.

## INTRODUCTION

Overweight and obesity are associated with increased incidence of cardiovascular and metabolic disease^1^. Treatments used to reduce body fat have included aerobic exercise, appetite suppressants, dieting, and lipase inhibitors^2^. The lack of success of these treatments has raised interest in other fat loss strategies, such as interval sprinting exercise (ISE)^3^ and green tea (GT) ingestion^4^.

GT, produced from the leaves of *camellia sinensis*^5^, contains a number of catechins, with the major catechins being epigallocatechin gallate (EGCG), epigallocatechin, epicatechin gallate (ECG), and epicatechin (EC)^6^. EGCG, which is the most pharmacologically active catechin^6^, has been shown to increase fat oxidation, particularly during the postprandial period, as indicated by a reduced respiratory quotient (RQ) during indirect calorimetry^7,8^. Although a mechanism underlying the GT and fat oxidation effect has not been determined it has been suggested that the catechins in GT inhibit catechol-O methyltransferase, an enzyme that degrades norepinephrine, which results in prolonging the action of sympathetically released norepinephrine^9^.

Venables et al.^10^ found that GT ingestion increased fat oxidation by 17%, compared with a placebo during a 30-minute continuous bout, of moderate intensity aerobic cycling exercise. It is conceivable that because steady state aerobic exercise results in small increases in plasma catecholamines^11^, other forms of exercise which induce a greater catecholamine response, may bring about a greater fat oxidation response after GT ingestion. For example, significantly elevated epinephrine and norepinephrine levels during 20 min of ISE in trained and untrained young women have been found^12^. This catecholamine response is an important characteristic of ISE as catecholamines have been shown to induce lipolysis and fat release from both subcutaneous and intramuscular fat stores^13^. Thus, increased catecholamine levels may explain why regular involvement in ISE induces greater fat loss than that after steady state exercise. For example, 15 weeks of ISE and steady state cycle exercise were compared, using a bout of sprinting exercise lasting 8 seconds, with a 12-second easy pedaling recovery (20 minutes total), for three times per week^14^. The total fat mass significantly decreased in the ISE condition whereas no reduction occurred after steady state exercise. A similar ISE protocol also resulted in significant decreases in visceral and total fat after 12 weeks of ISE in overweight males^15^ and females^16^.

Given the important role of the neurotransmitter norepinephrine in the control of fat oxidation, it is conceivable that GT, by inhibiting norepinephrine breakdown, may enhance fat oxidation after ISE. Support for this notion has come from a recent study that found that ingestion of GT elevated fat oxidation of young females after 20 minutes of ISE^17^. The plasma glycerol levels were also significantly elevated during ISE after GT ingestion, whereas epinephrine and norepinephrine levels were elevated during and after ISE. This study examined the effect of GT and ISE on fat oxidation of untrained females, however, whether this response is also present in aerobically trained and untrained males is undetermined. For example, it has been shown that catecholamine levels increase more in males than females during high-intensity exercise^18^. This response may be a result of the larger male muscle mass, which generates more power and enables men to typically work at a higher intensity than females.

The effect of one bout of ISE, with GT ingestion on fat oxidation after exercise, is important to establish as repeated use of this combination over months may reduce the fat mass of overweight individuals. Therefore, the major aim of this study was to examine the effect of a combination of GT and ISE on post-exercise fat oxidation. It was hypothesized that the combination of GT and ISE, compared with ISE alone, would result in significantly greater fat oxidation during the post-exercise recovery period for both the trained and untrained males.

## METHODS

### Participants

Twenty-eight healthy volunteer, non-smoking, aerobically trained and untrained males acted as participants. Their characteristics are shown in Table 1. The trained participants were cyclists and tri-athletes who had performed at least three exercise sessions per week for the last year at a moderate to hard exercise intensity. The trained participants were also required to attain a $\dot{V}O_{2max}$ of greater than 55 ml/kg/min. The untrained males were required to have no involvement in regular aerobic or anaerobic exercise for the last year. Exclusion criteria included men who were regular caffeine (≥ 2 cups coffee/day) or green tea drinkers (≥ 2 cups tea/day). The study was approved by a university human research ethics committee and all participants gave written informed consent.

**Table 1.** Characteristics of the participants (mean and SEM).

	Untrained		Trained	
	Mean	SEM	Mean	SEM
Age (yrs)	25.14	1.53	23.36	1.50
Height (cm)	177.00	2.53	178.00	1.12
Weight (kg)	80.53	3.78	73.39	1.67
BMI	25.66	1.02	23.16	0.40
$\dot{V}O_{2max}$ (ml/kg/min)	40.23	2.08	59.20	1.86
Total cholesterol (mmol/L)	4.06	0.23	3.86	0.17
HDL (mmol/L)	1.31	0.11	1.23	0.10
TG (mmol/L)	1.19	0.20	0.67	0.05
LDL (mmol/L)	2.22	0.17	2.10	0.18
TC/HDL	3.46	0.38	3.40	0.32
Glucose (mmol/L)	5.20	0.14	4.95	0.11
Resting heart rate (bpm)	59.00	1.31	55.00	2.60

### Preliminary testing

The participants arrived at the laboratory between 07:00 and 09:00 after an approximate 10-hour overnight NPO. Testing included baseline anthropometric measurements, fasting blood glucose level and lipid profile assessments, a$\dot{V}O_{2max}$ test, and ISE familiarization. Anthropometric measures included height, weight, and BMI. A 23-gauge butterfly needle (Terumo, Elkton, USA) was inserted into an antecubital vein and blood was placed in 4 ml heparin sodium and 10 ml EDTA vacutainers (Becton Dickinson, Plymouth, UK). Blood lipid levels were measured immediately, using whole blood (Cholestech LDX, Hayward, California, USA).

$\dot{V}O_{2max}$ was assessed using an electronically braked, computer-controlled Monark 839E ergometer (Monark, Vansbro, Sweden) connected to a metabolic cart (Parvo-Medics, Utah, USA). The participants maintained a cycling speed of 70 revolutions per minute (rpm) by pedaling to a metronome. After a 3-minute warm-up at 30 watts (W), the power output was increased by 15 W per min until exhaustion. The heart rate (HR) was recorded continuously, using a Polar Watch S810i (Polar Electro, Kempele, Finland). The laboratory was maintained at a constant ambient air temperature of 22°C to 23°C. The participants then practiced the ISE protocol on the Monark 839E ergometer.

### General study design

A double-blinded crossover counter-balanced design was used, which involved the trained and untrained men completing two exercise sessions with either the GT or the placebo. There was a wash-out period of a minimum of two weeks between the sessions.

### Diet and capsule content

Prior to the first session, the participants recorded a food diary for 3 days and were asked to follow the same diet before the second session. Twenty-four hours before each session, participants ingested one capsule containing either GT or cellulose with breakfast, lunch, and dinner. After an approximate 10-hour overnight NPO a fourth capsule was consumed in the morning, 90 minutes before exercising. GT catechins (EGCG, EGC, and EC) typically peak in the blood between 1.3 and 1.6 hours^19^. The GT capsule (GNC, Pennsylvania, USA) contained 250 mg of *camellia sinensis* extract (187.5 mg polyphenols, 125 mg EGCG, and 20 mg caffeine), whereas the placebo capsule contained 500 mg cellulose^20^. All participants were reminded by text message to ingest the GT capsules the day before and on the morning of exercise.

### Experimental protocol

The second and third exercise sessions, which were counterbalanced, consisted of: 1) rest, 2) ISE, and 3) post-exercise recovery. Participants arrived at the laboratory between 07:00 and 09:00 a.m. after the approximate 10-hour NPO, then a 22-gauge cannula (Becton Dickinson, Plymouth, UK) was inserted into an antecubital vein. A 3-way stopcock (Becton Dickinson, Plymouth, UK) was used for repeated blood sampling. The line was kept patent by flushing with 0.9% isotonic saline (Pfizer, New York, USA). A baseline blood sample was collected 40 minutes after insertion of the cannula. The blood samples were taken at rest (baseline), during, and post-exercise and were collected in EDTA vacutainers and then centrifuged for 10 minutes at 3000 rpm. Blood plasma was then extracted and stored at -86°C for glycerol and catecholamine analysis.

### Rest

Each participant rested supine for 10 min before RQ assessment. The RQ was measured when the HR and breathing rhythm were stable, whereas the HR was monitored continuously. The RQ was measured for 30 min using a canopy system (True One 2400 ParvoMedics, Utah, USA). At the end of the rest period the blood lactate levels were measured, using whole blood obtained from the indwelling cannula (Accutrend Lactate monitor, Roche, Germany).

### Interval sprinting exercise

Participants warmed up for 5 min at 30 W, and then completed 20 min of ISE on a Monark Ergomedic 839E ergometer connected to a metabolic cart (ParvoMedics, Utah, USA) at 65% of their maximal power output during the 8-s sprint phase, and at approximately 25% of maximal power output during the 12-s easy pedaling recovery phase. During the 20-min ISE session, sixty 8-s/12-s bouts totaling 8 min of sprinting and 12 min of recovery were performed followed by a 5-min cool-down at 30 W. The HR was monitored continuously, whereas rpm was recorded for every sprinting interval. Exercise was accompanied by a tape, which prompted the start and finish and the pedal cadence (115 rpm) during the sprint and recovery (40-44 rpm) phases.

The blood was sampled at 7, 14, and 20 minutes during exercise and blood samples from minutes 7 and 20 were assayed immediately for lactate. Glycerol levels were assessed from blood collected before exercise; 7, 14, and 20 minutes during exercise; and 15, 30, 45, 60, and 75 minutes post-exercise. Epinephrine and norepinephrine levels were assessed from blood collected before exercise; after 20 minutes of ISE; and 15 minutes post-ISE. The RER was averaged at 5-minute stages but was only analyzed during the 30 to 75 minute post-exercise because the changing blood lactate concentration (0 to 30 minutes after exercise) also changed the bicarbonate concentration, which results in CO_2_ production that influences RER without necessarily representing the true quotient21. Lactate levels were stable during the period 30 to 75 minutes post-exercise.

### Post-exercise

The participants rested for 10 minutes post-exercise while gas and volume calibration were undertaken, after which 75 minutes of post-exercise recovery under the ventilated canopy was performed. Blood was sampled every 15 minutes for 75 minutes post-exercise, and was assayed immediately for lactate at 30 and 60 minutes post-exercise. HR was recorded continuously and was averaged every 15 seconds.

### Blood variables and fat oxidation rate

The Free Glycerol Determination (FG0100) reagent assay kit (Sigma Aldrich) and Glycerol Standard (G7793) were used to assess glycerol. Dual wavelength absorbance measurements at 450 nm and 540 nm were used to determine the degree of enzymatic turnover of the substrate. The coefficient of variation (CV) was 7.8%. The norepinephrine and epinephrine levels were measured by means of mass spectrometry with a 5973N Mass Selective Detector, coupled to a 6890N gas chromatograph, and an SGE Forte BPX5 x 0.25 IDx 0.25 micron column22. The accuracy and precision were determined by analysis of spiked serum samples at low, medium, and high nM concentrations of epinephrine and norepinephrine in triplicate on 7 separate days. Serum epinephrine recoveries at 2, 10, 50 nM were above 99% and the inter-day average CV was 5.88%, whereas norepinephrine recoveries at 20, 100, and 500 nM were above 95% and the inter-day average CV was 4.28%. The average intra-day CV was 2.26% for epinephrine and 2.02% for norepinephrine. The average intra-day recoveries of 102% and 98.6% were obtained for epinephrine and norepinephrine, respectively.

Fat and carbohydrate oxidation rates (g/min) were calculated using the following equations [23]:

(1) $$Fat\;oxidation\;\left(1.695\;x\;\dot{V}O_{2}\right)-(1.701\;x\;\dot{V}{CO}_{2})$$

(2) $$Carbohydrate\;oxidation\;\left(4.585\;x\;\dot{V}O_{2}\right)-(3.226\;x\;\dot{V}{CO}_{2})$$

When assessing fat oxidation rate, only the values at rest and during minutes 30 to 75 during exercise recovery, were used because RER does not represent substrate utilization when blood and tissue lactate concentrations are changing^21^.

### Statistical analysis

Data were analyzed using SPSS 20.0 (SPSS Inc, Chicago, IL). A two-factor (time x condition) repeated measures ANOVA was used to compare differences across time, condition, and group. The Mauchly’s sphericity test was employed to examine the homogeneity of covariance for within subject factors. The Greenhouse-Geisser test was used to correct for non-homogenous values. With repeated measures, when ANOVA interactions were significant, adjusted Bonferroni post hoc tests were also performed. Data are presented as mean ± SEM and significance was set at p < 0.05.

## RESULTS

### Participant characteristics

Body composition, age, $\dot{V}O_{2max}$, lipids, glucose, and resting HR are shown in Table 1.

### Workload and exercise intensity

There were no significant differences between the two conditions in the mean power output, RPE, lactate levels, rpm (Table 2), and HR (Table 3). The untrained compared with the trained males, however, recorded significantly less mean power output, and had greater HR and lactate levels in both conditions (Tables 2 and 3).

**Table 2.** Characteristics of the participants (mean and SEM) response of trained and untrained males to the interval sprinting exercise during the placebo and green tea conditions (mean and SEM).

	Trained		Untrained	
	Placebo	GT	Placebo	GT
Mean power output (W) during 8 s sprint	213 ± 5.3	213 ± 5.3	173 ± 10.3#	173 ± 10.3*
Revolutions per minute during 8 s sprint	115.3± 0.6	115.5± 0.8	115.1±1.2	116.2± 1.0
Pedal resistance (kg) during 8 s sprint	1.85± .10	1.85± .11	1.5± .10#	1.5± .09*
Rating of perceived exertion throughout	13.4± 0.5	13.6± 0.4	13.3± 0.5	13.5± 0.3
Lactate (mmol/L) at 7 min	3.7± 0.3	3.6± 0.3	4.1± 0.3#	3.9± 0.3*
Lactate (mmol/L) at 20 min	3.9± 0.4	3.9± 0.3	5.2± 0.5#	5.2± 0.4*

GT : green tea; *significantly different between groups for the GT condition, p < 0.05

# significantly different between groups for the placebo condition, p < 0.05

**Table 3.** Heart rate and $\dot{V}O_{2}$ response of the trained and untrained males before, during, and after interval sprinting in the green tea and placebo conditions (mean and SEM).

Variable	Condition	Rest	Exercise	Post-exercise			
				15 minutes	30 minutes	45 minutes	60 minutes
Heart rate (bpm) Trained	GT	54± 3*	143± 4	66± 3	61± 2	58± 2	56± 2
	Placebo	56± 3#	140± 3	67± 3	62± 3	58± 2	56± 2
Heart rate (bpm) Untrained	GT	60± 1*	151± 4	78± 3	72± 3	70± 3	71± 3
	Placebo	60± 1#	150± 4	79± 2	73± 2	70± 2	70± 2
VO_2_ (ml/kg/min) Trained	GT	3.9± 0.1	35.3± 1*	4.0± 0.1	3.8± 0.1	3.7± 0.2	3.8± 0.2
	Placebo	3.8± 0.1	35.3± 1#	4.0± 0.1	3.8± 0.1	3.7± 0.2	3.7± 0.1
VO_2_ (ml/kg/min) Untrained	GT	3.5± 0.1	27.6± 1.4*	3.9± 0.1	3.7± 0.1	3.6± 0.1	3.7± 0.1
	Placebo	3.4± 0.1	27.8± 1.3#	3.8± 0.1	3.6± 0.1	3.4± 0.1	3.6± 0.1

GT : green tea; *significantly different between groups for the GT condition, p < 0.05

# significantly different between groups for the placebo condition, p < 0.05

### Respiratory exchange ratio(RER) and fat oxidation

**Figure 1.**
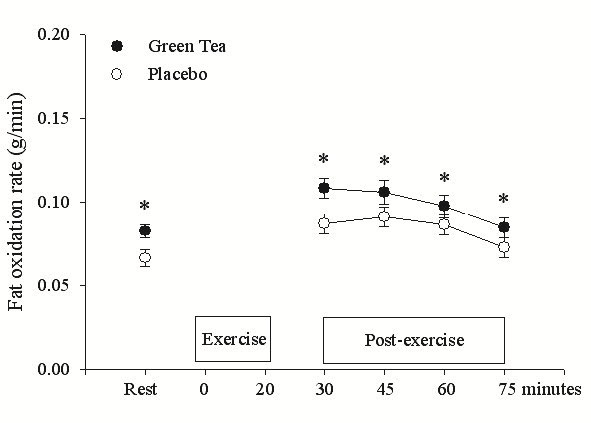
Fat oxidation at rest and after interval sprinting exercise with either placebo or green tea ingestion for the trained and untrained males combined. *significantly greater than the placebo, p < 0.05.

During the resting period, before exercise, RER was significantly decreased, p < 0.05, after GT ingestion (0.82 ± 0.08) compared with the placebo (0.86 ± 0.08). There was also a significant condition main effect (p < 0.01) with RER being significantly decreased from minutes 30 to 75 post-exercise in the GT (0.79 ± 0.07) compared with the placebo condition (0.82 ± 0.01). During the resting period, before exercise, fat oxidation significantly increased by 20% for the untrained and trained groups (p < 0.01) after GT ingestion compared with the placebo (Figure 1). In the 30 to 75 min period during the post-exercise period there was a significant condition main effect (p < 0.01) with the fat oxidation rate being significantly increased by 15% from minutes 30 to 75 in the GT condition, compared with the placebo (Figure 1). The fat oxidation was significantly greater in the resting condition after green tea ingestion (p < 0.05), compared with the placebo.

### Glycerol and catecholamine levels

**Figure 2.**
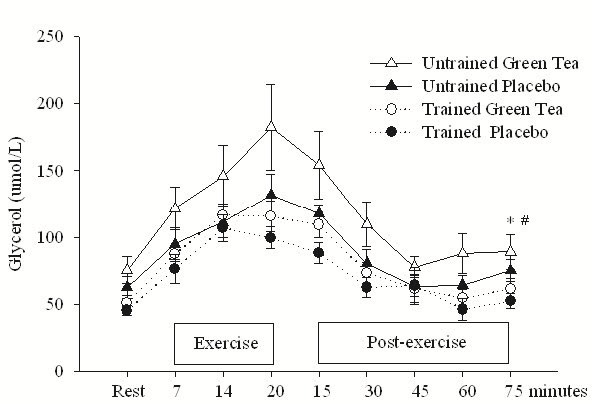
Glycerol levels at rest, during, and after interval sprinting exercise with either placebo or green tea ingestion for the trained and untrained males. *trained and untrained combined significantly greater in the green tea condition compared with the placebo during rest, exercise, and post-exercise, p < 0.05; #untrained significantly greater than the trained in the green tea condition, p < 0.05.

**Figure 3.**
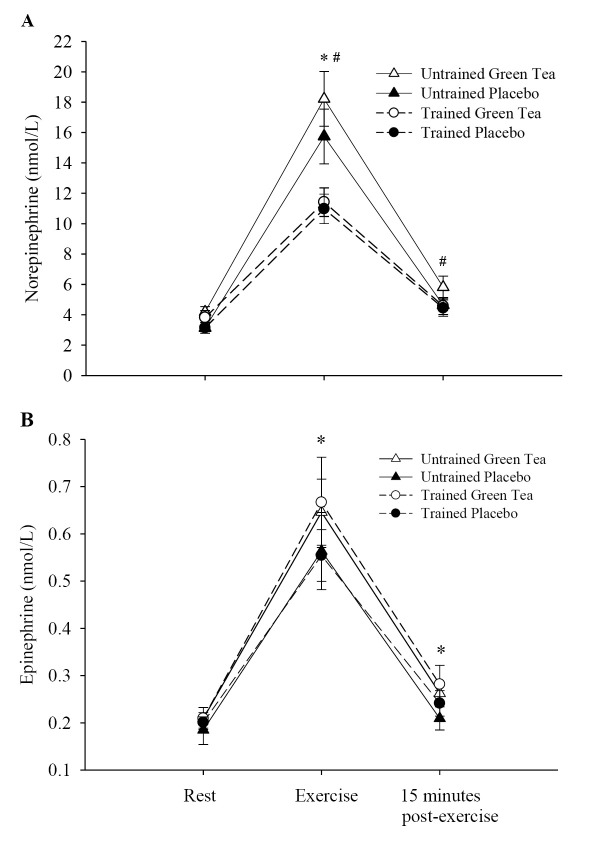
Norepinephrine (a) and epinephrine (b) levels at rest, during, and after interval sprinting exercise with either placebo or green tea ingestion for the trained and untrained males. (a) *untrained significantly greater than the trained in the green tea and the placebo conditions, p < 0.05; #untrained significantly greater in the green tea condition compared with the placebo, p < 0.05; (b) *trained and untrained significantly greater in the green tea condition compared with the placebo during and after interval sprinting exercise, p < 0.05.

Compared with the baseline, both groups combined possessed significantly increased glycerol levels during exercise in the GT condition, p < 0.5. There was also a significant group effect, p < 0.05, with the untrained group recording significantly greater glycerol concentration during and post-exercise, compared to the trained group (Figure 2). GT ingestion brought about significantly greater norepinephrine concentration during and after exercise in the GT condition for only the untrained group (Figure 3a). The plasma epinephrine concentration compared with the placebo for both groups, was significantly higher (p < 0.05) during exercise and post-exercise (Figure 3b).

### $\dot{V}O_{2}$

$\dot{V}O_{2}$ was similar during rest, exercise, and post-exercise in both the GT and placebo conditions for the trained and untrained (Table 3). $\dot{V}O_{2}$ was significantly higher, p < 0.05, for the trained males during ISE (Table 3).

## DISCUSSION

The combined effect of GT ingestion and ISE on fat oxidation of the trained and untrained males was assessed. During pre-exercise rest, the GT ingestion resulted in a significant increase in fat oxidation. After GT ingestion, the fat oxidation was significantly greater throughout the 30 to 75 minute intervals of the post-exercise period. The plasma glycerol and epinephrine levels during and after exercise for both groups were significantly higher after GT consumption, compared with the placebo condition. Plasma norepinephrine levels were significantly higher after GT consumption, compared with the placebo condition, during and after exercise for the untrained group only. No significant differences existed between the trained and untrained groups for fat oxidation, however, glycerol and norepinephrine was greater in the untrained males.

The trained participants in this study were cyclists and tri-athletes who had performed at least three exercise sessions per week for the last year at a moderate to hard exercise intensity. That they possessed low resting HR and above average $\dot{V}O_{2max}$ (Table 1) supports the notion that they were aerobically trained. Their quicker HR recovery response after ISE and their lower lactate levels during ISE also indicate a trained status and that they were better adapted to this form of interval sprinting exercise even though none had carried out this kind of training previously. Their fat oxidation response to both GT and ISE, however, was similar to that of their untrained counterparts. When fat oxidation response was adjusted for the smaller body mass of the trained, fat oxidation was still similar to that of the untrained male group.

The trained group recorded higher $\dot{V}O_{2}$ and lower lactate and HR during ISE. The untrained group, however, recorded significantly higher glycerol levels during ISE, compared with the trained group. Also norepinephrine levels during exercise were higher in the untrained group compared with the trained group. These differences may have been related to the HR and lactate response of the untrained, even though both groups exercised at the same relative intensity. The lower HR and lactate response of the trained indicates that they were better adapted to ISE. Interestingly, despite having greater lactate levels during exercise, fat oxidation after exercise was slightly higher in the untrained male group, thereby suggesting that glycolysis did not have a significant inhibitory effect on fat oxidation.

That ingestion of GT increases fat oxidation at rest has been demonstrated by previous studies. Dulloo et al.^7^ found that GT increased resting fat oxidation by 10%, whereas Rumpler et al.^24^ recorded a 12% increase. Gahreman et al.^17^ examined females and showed a greater fat oxidation increase during rest, after GT ingestion of 24%. In the present study the untrained and trained male groups recorded a fat oxidation increase during rest of 20% after GT ingestion (Figure 1). These results demonstrate that GT consumption also significantly elevates resting fat oxidation rate of the trained and untrained groups. Similar to women^12^, the higher plasma glycerol levels at rest also indicate that GT, compared with the placebo, resulted in enhanced lipolysis.

The sprinting component (fast pedaling) of ISE is primarily fuelled by creatinine-phosphate and anaerobic glycolysis^25^. Oxygen bound to myoglobin also makes a minor contribution to energy production during sprinting. The major role of aerobic metabolism, however, appears to be the resynthesis of creatinine-phosphate during the sprint recovery periods^25^. Intramuscular triglyceride (IMTG) stores are likely to be the major source of fat oxidation during ISE as subcutaneous adipose fat stores make only a small contribution during high-intensity exercise^26^. The glycogen depletion, occurring through continuous sprinting, is likely to impede glycolysis, thereby resulting in increased oxidation of IMTG^26^. During intense sprinting exercise glycogen stores suffer greater depletion than steady state exercise^27^. Consequently, the recovery period, after ISE has ended, should involve enhanced lipid oxidation so that the remaining carbohydrates can be used for glycogen resynthesis^21^. These results and those of the study by Gahreman et al.^17^ support the notion that for the placebo condition, greater fat oxidation occurred throughout the post-exercise period for both men and women. The enhanced glycerol and catecholamine response to ISE appears to be a feature of this kind of exercise^12^, and may have implications for longterm fat loss^3^.

It was hypothesized that enhanced fat oxidation would occur after the ingestion of GT during the post-exercise period. Some factors underlying the increase in post-exercise fat oxidation are enhanced norepinephrine and epinephrine release during ISE, thus resulting in a possible increase of circulatory sulfo-conjugated catecholamines^28^. As discussed previously, it is thought that GT catechins increase fat oxidation through the inhibition of catechol-O methyltransferase, the enzyme that degrades norepinephrine, thereby prolonging the adrenergic drive^9^. This hypothesis is supported by the increased catecholamine and glycerol levels during and after exercise after GT ingestion, together with the enhanced fat oxidation.

With regard to gender differences when fat oxidation was adjusted to body mass, there was a similar response of both untrained women^17^ and men (present study) to ISE and GT. The effect of gender, on fat oxidation after ISE, has not been directly investigated, however, the fat oxidation response of males and females during aerobic exercise has been examined. Unfortunately, studies have produced equivocal results. For example, some studies using microdialysis probes or isotope tracers found that the lipolytic rates of females were greater than that of males during aerobic exercise^29^. In contrast, others have shown that the lipolytic response to exercise is the same for both genders^30^. Studies using indirect calorimetry have also demonstrated that men oxidize less fat and carbohydrate than women during aerobic exercise^31^. The equivocality of these results may have been brought about by differences in aerobic fitness levels, body composition, and exercise modality^32^. In the study by Gahreman et al.^17^ women possessed an average $\dot{V}O_{2max}$ of 32 ml/kg/min which was smaller than the 40 ml/kg/min of the untrained males of the present study. Despite these aerobic fitness differences, when adjusted for body mass, there was a similar post-exercise fat oxidation for these women and men.

Glycerol levels, however, were higher during and postexercise for women^17^, compared with men, of the present study. This response suggests that after ISE, the free fatty acid (FFA) availability was greater in women than in men. The greater glycerol levels, indicating higher levels of plasma fatty acids, suggest that women oxidized more FFA from adipose tissue triglycerides and less non-plasma FFA that is derived from IMTG^13^. However, the underlying mechanisms are unknown, concerning these possible gender differences regarding lipolysis of adipose tissue triglycerides^33^. The exercise-induced increase in lipolytic activity is primarily driven by the stimulation of adipose tissue β-adrenergic receptors by circulating catecholamines. Activation of α-adrenergic receptors, which inhibit lipolysis, also influences the lipolytic response to exercise^34^. The plasma catecholamine concentration during exercise was similar in the male and female participants^17^; thus, the higher rate of lipolysis in women was likely caused by a combination of increased adipose tissue sensitivity to β-adrenergic stimulation and decreased adipose tissue sensitivity to α-adrenergic stimulation.

Each green tea capsule contained 20 mg of caffeine, however, a single oral dose in excess of 100 mg caffeine is needed to bring about a significant increase in thermogenesis7. To produce an increase in energy expenditure, a 600 to 1000 mg caffeine dose per day has been suggested^35^. Thus, the 20 mg of caffeine ingested by participants in the current study is unlikely to have had a significant effect on resting and exercise fat oxidation levels. With regard to post-aerobic exercise fat oxidation and the length of monitoring, Mulla et al.^36^ examined adipose tissue and skeletal muscle lipid metabolism after 60 min of easy (40% $\dot{V}O_{2max}$) and hard (60% $\dot{V}O_{2max}$) aerobic exercise and found that there was a quick decrease in lipolysis immediately after the cessation of exercise which increased about 1 hour post-exercise and remained elevated for the following 2 hours. The increase in post-exercise non-esterified fatty acids mobilization was greater after 60% exercise than after 40% exercise. Thus, it is possible that there may be a greater lipolytic response to ISE hours after exercise. Consequently, there is a need for studies monitoring metabolic and hormonal response to ISE for an extended period.

A limitation of this study was that the blood levels of the different catechins contained in the GT were not assessed, thus, it cannot be shown that GT directly influenced fat oxidation. Also, another limitation concerns the assessment of the active nutrient content contained in the green tea capsules, which was not independently analyzed.

In conclusion, it was found that GT significantly increased fat oxidation before and post-exercise. Epinephrine levels were elevated during and post-exercise after GT consumption, for both the trained and untrained groups. The exercise and post-exercise levels of norepinephrine levels, after green tea ingestion, were elevated for the untrained group only. The plasma glycerol levels were also significantly elevated during exercise after the GT ingestion compared with the placebo condition. However, the untrained group, compared with the trained group, recorded a larger glycerol and norepinephrine response during ISE after ingestion of GT. The significant increase in fat oxidation caused by one bout of ISE, together with GT ingestion, suggests that repeated use of this combination over months may reduce the fat mass of overweight males.
